# Supplementary material for: Gradual Expansion of a Stent to Prevent Periprocedural Complications after Carotid Artery Stenting for Vulnerable Severe Stenotic Lesions with Intraplaque Hemorrhages: A Retrospective Observational Study
Source: Life (Basel). 2022 Jan 17;12(1):131. doi: 10.3390/life12010131 (PMC8781376; doi:10.3390/life12010131)
Supplement: Supplementary file 1 [file life-12-00131-s001.zip › life-1542007-supplementary.pdf]

life

MDPI

Article

Supplementary Materials: Gradual Expansion of a Stent to Prevent Periprocedural Complications after Carotid Artery Stenting for Vulnerable Severe Stenotic Lesions with Intraplaque Hemorrhages: A Retrospective Observational Study

Table S1. Information about procedures of carotid artery stenting.

Groups	Overall	IPH group	non-IPH group
	N = 68	N = 30	N = 38
**CAS procedure**		**GE**	**standard**
Lesion laterality			
Right, n (%)	36 (52.9%)	19 (63.3%)	11 (36.7%)
Approach for introducing a stent	Transbrachial	Transbrachial	Transbrachial
Distal protection during CAS	Filter	Filter	Filter
Proximal protection during CAS	None	None	None
Balloon diameter for pre-SA			
3.0 mm, n (%)	22 (32.4%)	22 (66.7%)	0
4.0 mm, n (%)	46 (67.6%)	8 (33.3%)	38 (100%)
Inflated balloon diameter (mm)		3.0 or 4.0 mm	4.0 or 4.23 mm
Balloon inflation pressure	8 or 14 atm	8 atm (NP)	14 atm except for ex-CS
Implanted stents			
Closed-cell stent, n (%)	68 (100%)	30 (100%)	38 (100%)
Nominal diameter of the implanted stent, mm	10.0 mm	10.0 mm	10.0 mm
Post-SA	No	No	No
Procedural time, (minutes), median (IQR)	57 (46, 67)	52 (44, 67.5)	60 (53, 68)

CAS, carotid artery stenting; ex-CS, extremely high-grade carotid stenosis; GE, gradual expansion; IPH, intraplaque hemorrhage; IQR, interquartile range; n, number; NP, nominal pressure; SA, stenting angioplasty.

Table S2. Minimal luminal diameter before carotid artery stenting and minimal stent diameter immediately after carotid artery stenting.

	MLD before CAS	MSD after CAS	*p*-value
Overall (n=68), mm	0.85 (0.54, 1.09)	3.42 (2.82, 3.73)	< 0.001
IPH group (n=30), mm	0.76 (0.54, 1.05)	2.97 (2.62, 3.54)	< 0.001
non-IPH group (n=38), mm	0.90 (0.53, 1.14)	3.58 (3.13, 3.86)	< 0.001

All values are represented as median (interquartile range). CAS, carotid artery stenting; IPH, intraplaque hemorrhage; MLD, minimal luminal diameter; MSD, minimal stent diameter; n, number; p, probability.

Table S3. Carotid artery stenosis rate before and immediately after carotid artery stenting.

	CASr before CAS	CASr after CAS	*p*-value
Overall (n=68), %	80.9 (76.9, 86.7)	29.2 (18.6, 38.8)	< 0.001
IPH group (n=30), %	81.2 (77.4, 88.1)	37.5 (26.6, 44.6)	< 0.001
non-IPH group (n=38), %	80.7 (76.5, 86.0)	26.4 (15.9, 34.2)	< 0.001

All values are represented as median (interquartile range). CAS, carotid artery stenting; CASr, carotid artery stenosis rate; IPH, intraplaque hemorrhage; n, number, p, probability.

Table S4. Middle cerebral artery relative signal intensity before and immediately after carotid artery stenting.

	MCA rSI before CAS	MCA rSI after CAS	*p*-value
Overall (n=68)	0.87 (0.74, 0.98)	1.01 (0.92, 1.06)	< 0.001
IPH group (n=30)	0.88 (0.74, 0.98)	1.02 (0.97, 1.06)	< 0.001
non-IPH group (n=38)	0.87 (0.74, 0.96)	0.98 (0.89, 1.07)	< 0.001

All values are represented as median (interquartile range). CAS, carotid artery stenting; IPH, intraplaque hemorrhage; MCA, middle cerebral artery; n, number; p, probability; rSI, relative signal intensity.

Table S5. Peak systolic velocity before and immediately after carotid artery stenting.

	PSV before CAS	PSV after CAS	*p*-value
Overall (n=68), cm/s	281 (210, 348)	83 (67.2, 113.8)	< 0.001
IPH group (n=30), cm/s	271 (215, 337)	97.6 (70.0, 147)	< 0.001
non-IPH group (n=38), cm/s	293 (178, 361)	80.1 (64.9, 91.2)	< 0.001

All values are represented as median (interquartile range). CAS, carotid artery stenting; IPH, intraplaque hemorrhage; n, number; p, probability; PSV, peak systolic velocity.

Table S6. Minimal stent diameter immediately after and at 4 months after carotid artery stenting.

	MSD after CAS	MSD at 4 months after CAS	*p*-value
Overall (n=59), mm	3.26 (2.64, 3.80)	3.97 (3.46, 4.47)	< 0.001
IPH group (n=26), mm	2.97 (2.62, 3.54)	3.89 (3.22, 4.75)	< 0.001
non-IPH group (n=33), mm	3.58 (3.13, 3.86)	4.04 (3.57, 4.33)	< 0.001

All values are represented as median (interquartile range). CAS, carotid artery stenting; IPH, intraplaque hemorrhage; MSD, minimal stent diameter; n, number; p, probability.

Table S7. Carotid artery stenosis rate immediately after and at 4 months after carotid artery stenting.

	CASr after CAS	CASr at 4 months after CAS	*p*-value
Overall (n=59), %	28.1 (18.4, 38.8)	26.0 (16.5, 33.5)	< 0.001
IPH group (n=26), %	37.5 (26.6, 45.9)	27.7 (18.5, 40.7)	< 0.001
non-IPH group (n=33), %	25.9 (16.0, 34.0)	24.3 (14.2, 32.2)	< 0.001

All values are represented as median (interquartile range). CAS, carotid artery stenting; CASr, carotid artery stenosis rate; IPH, intraplaque hemorrhage; n, number; p, probability.
